# Performance of Primary Care Physicians in the Management of Glycemia, Lipids, and Blood Pressure among People with Type 2 Diabetes: A Cross-Sectional Study

**DOI:** 10.3390/jcm13061544

**Published:** 2024-03-07

**Authors:** Bogdan Vlacho, Berta Fernandez-Camins, Albert Canudas-Ventura, Andrés Rodríguez, Àngels Mollo, Francesc Xavier Cos Claramunt, Maria Antentas, Dídac Mauricio, Josep Franch-Nadal

**Affiliations:** 1 DAP-Cat Group, Unitat de Suport a la Recerca Barcelona, Fundació Institut Universitari per a la Recerca a l’Atenció Primària de Salut Jordi Gol i Gurina (IDIAPJGol), 08007 Barcelona, Spain; bogdan.vlacho@gmail.com (B.V.); fernandezcamins@gmail.com (B.F.-C.); angelsmollo@gmail.com (À.M.); francescxaviercos@gmail.com (F.X.C.C.); 2 Department of Endocrinology & Nutrition, Hospital de la Santa Creu i Sant Pau, IR Sant Pau, 08025 Barcelona, Spain; maantentas@gmail.com; 3 CIBER of Diabetes and Associated Metabolic Diseases (CIBERDEM), Instituto de Salud Carlos III (ISCIII), 28029 Madrid, Spain; 4 Department of Medicine, University of Lleida, 25008 Lleida, Spain; albertcanudas5@gmail.com; 5 Primary Health Care Center Vall del Tenes, Gerència d’Àmbit d’Atenció Primària Metropolitana Nord, Institut Català de la Salut, 08028 Barcelona, Spain; 6 Primary Health Care Center Tàrrega, Gerència d’Àmbit d’Atenció Primària Lleida Ciutat, Institut Català de la Salut, 25007 Lleida, Spain; arodga@gmail.com; 7 Innovation Office at Institut Català de la Salut, 08007 Barcelona, Spain; 8 Department of Medicine, University of Vic—Central University of Catalonia, 08500 Vic, Spain; 9 Primary Health Care Center Raval Sud, Gerència d’Àmbit d’Atenció Primària Barcelona Ciutat, Institut Català de la Salut, 08001 Barcelona, Spain

**Keywords:** T2DM, inter-professional variability, primary health care professionals, glycemic control, lipids, blood pressure

## Abstract

**Background:** Our study aimed to evaluate the performance of primary healthcare physicians (PCPs) in managing glycemia, lipids, and blood pressure in people with type 2 diabetes mellitus (T2DM) in Catalonia, Spain. **Methods:** We included 3267 PCPs with 367,132 T2DM subjects in a cross-sectional analysis of the SIDIAP (Sistema d’Informació per al Desenvolupament de la Investigació en Atenció Primària) database for the year 2017. **Results**: 63.1% of PCPs were female, with an average practice size of 1512 subjects. T2DM individuals had a mean (standard deviation) age of 70 (±12.2) years old, a mean body mass index (BMI) of 30.2 (±5.21) kg/m^2^, and a median diabetes duration of 8.8 years. Overall, 42.6% of subjects achieved target glycemic control (glycated hemoglobin < 7%). Notably, 59.2% maintained blood pressure < 140/90 mmHg during the 12-month study period. The multivariable analysis identified positive associations between glycemic control and female PCPs, practice sizes (1000–1500 people), a higher proportion of patients aged ≥ 65 years, and rural practices. Combined glycemic, lipid, and blood pressure target attainment was associated with medium-sized practices and those with a higher proportion of patients aged ≥ 65 years. **Conclusions:** Practice size, patient age distribution, and rurality are factors associated with the performance of PCPs in the control of glycemia, lipids, and blood pressure in T2DM subjects in primary health care centers in our region.

## 1. Introduction

Type 2 diabetes mellitus (T2DM) is a highly prevalent chronic metabolic disorder that poses a substantial global health challenge associated with considerable morbidity and mortality. The prevalence of T2DM is continuing to rise at an alarming rate, predicted to affect 7079 individuals per 100,000 by 2030, mainly attributed to changes in lifestyle factors such as diet and activity levels [1,2]. Importantly, T2DM is a significant risk factor for cardiovascular disease, both by itself and by its association with other risk factors, such as dyslipidemia or high blood pressure [3]. In the past two decades, as a consequence of advances in new therapies and multifaceted management of cardiovascular risk factors, important reductions in the mortality and incidence of cardiovascular complications among people with T2DM have occurred. However, cardiovascular complications remain the leading cause of death and decrease in quality of life among people with T2DM [4]. 

For a reduction in the risk of diabetes complications (micro and macrovascular), most current clinical guidelines recommend a multifactorial approach, focusing on the proper management of glycemia, lipids, blood pressure, and the use of drugs with proven cardiovascular and renal benefits [5,6]. However, evidence suggests that glycemic and cardiovascular risk factor (CVRF) control in different countries is far from optimal and has worsened over the years among people with T2DM [7]. For example, the results of the national ELIPSE study concluded that the degree of control of different CVRF among people with T2DM in different primary care centers does not guarantee adequate cardiovascular prevention; this showed the need for more intensive use of pharmacological treatment [8]. Real-world studies from our country have further highlighted insufficiencies in achieving the recommended goals of disease control [9,10]. In our region of Spain (Catalonia, Northeast Spain), approximately 45% of people with T2DM do not achieve the glycated hemoglobin (HbA1c) target levels, about 30% have inadequate blood pressure control, and half of them have poor lipid control [11]. The reasons for suboptimal glycemic and CVRF control are diverse and related to the subjects with T2DM (lack of adherence to the treatment and poor health awareness) [12] and healthcare professionals (therapeutic inertia and lack of resources or experience) [13,14]. 

According to recent recommendations, managing T2DM should be patient-centered, considering the patient’s needs, preferences, and values, and involving collaborative and multidisciplinary healthcare teams from different levels [15,16]. Primary healthcare professionals are usually the first line of health care for managing people with T2DM in many healthcare settings [17,18], and they play a vital role in patient care and outcomes. However, the healthcare for people with T2DM is often carried out in isolation, leading to heterogeneity in practice patterns [19], including differences in the implementation, experience, and use of decision tools. This variability encompasses diverse clinical strategies, treatment recommendations, and patient interactions, raising questions about its implications for patient outcomes and healthcare quality. Different studies have shed light on the extent of this variability within diabetes care [20,21]. Research conducted in Switzerland by Peytremann-Bridevaux et al. described patients’ and healthcare professionals’ perceptions regarding the difference in the quality of diabetes care [22]. Patients referred to differences in the healthcare consultation time and information received by primary care physicians (PCPs) and specialists. Meanwhile, PCPs pointed out the lack of follow-up, communication, and collaboration among the professionals to achieve better-quality diabetes care. Another study by Simmons et al. in New Zealand reported important differences in perceptions of barriers (psychological, motivational, self-efficacy, educational, physical, and psychosocial) in diabetes care between healthcare professionals [23]. However, there is limited research on the performance of PCPs in managing CVRFs, including glycemia, lipids, and blood pressure, among people with T2DM.

Overall, understanding the factors contributing to the performance of PCPs in their daily clinical practice is essential for optimizing diabetes care and standardizing management approaches. For this reason, we conducted the current study to investigate the performance among the PCPs in achieving control values of the most common cardiovascular risk factors among people with T2DM in Catalonia and factors influencing the performance.

## 2. Materials and Methods

### 2.1. Study Design and Settings 

We performed a cross-sectional study using retrospective, routinely collected primary healthcare data from the Barcelona, Spain, SIDIAP (Sistema d’Informació per al desenvolupament de la Investigació en Atenció Primària) database in the period from 1 January 2017 to December 2017. The SIDIAP database has been previously used for different epidemiological studies, and it is a well-validated primary healthcare database for research on diabetes and its complications [24,25]. This population database includes routinely collected healthcare information from people attending the primary healthcare centers at the Institut Català de la Salut (ICS). 

ICS is the largest healthcare provider in Catalonia. The healthcare system in Catalonia is publicly funded, with universal access for all citizens. Primary care centers serve as the main point of contact for individuals with health concerns, including diagnosing and managing T2DM. Antidiabetic treatment is provided free of charge for retired and severely ill individuals, while employed individuals, regardless of income level, contribute only a small percentage of drug costs. For the study, 288 primary healthcare teams were available, providing nearly 40 million visits to primary care centers annually and approximately 84,125 daily visits with PCPs [26]. For the study, two data sources were merged and linked: the database of subjects who attended the primary care centers and the details on PCPs providing care for those subjects. 

The study was reviewed and approved by Primary Health Care University Research Institute Jordi Gol, Barcelona, Spain (initial approval number P13/014 on 27 May 2013, modified and approved on 14 May 2018).

### 2.2. Study Subjects

All active PCPs were identified, and small-sized practices (less than 400 patients) were excluded from the analysis due to the possibility of errors in the database or not being truly involved in providing full-time healthcare. From the remaining PCPs, we included all the active subjects aged between 30 and 90 years with a diagnosis of T2DM in the database, defined as the presence of ICD10 diagnostic codes E11 or E14. All the subjects with other types of diabetes (type 1, gestational, or other causes), death, withdrawal from the database, or recent diagnosis (less than one year of disease duration) were excluded from the analysis.

### 2.3. Variables and Definitions

At the observational period end date (December 2017), for PCPs, variables related to sex, practice size (number of patients in the practice), proportion of patients at least 65 years old, deprivation index (MEDEA) where the primary health care centers were located, and rurality (types: urban, semi-urban, and rural area) were collected. The MEDEA deprivation index (DI) assesses five socioeconomic indicators related to work and education (i.e., unemployment, manual and eventual workers, and insufficient education overall and in young people) extracted from census tracks [27]. It detects small areas of large cities in Spain with unfavorable socioeconomic characteristics and is associated with overall mortality. The higher the DI, the worse the social deprivation is. For rurality, the classification or categorization of areas is based on their level of urbanization or rural characteristics. The urban and rural areas differ regarding healthcare accessibility, characteristics, and resources available [28,29,30,31]. This classification helps understand how healthcare data might vary across different types of areas, such as urban, suburban, and rural regions. Moreover, the healthcare quality standard (EQA) and the Pharmaceutical Prescribing Quality Standard (EQPF) variables were collected. Since 2006, the EQA tool has been used in primary care centers of ICS to measure, through indicators (out of a total of 60), the adequate management of different conditions and the implementation of preventative activities [32]. The EQPF is a measurement tool used since 2003 in the professional incentive process to improve the quality of medication prescriptions in primary care. The EQPF gathers all the scientific evidence for the drugs recommended by the ICS. These drugs correspond to the eleven groups of medicines used to treat the most common health problems in primary care, and the origin of the prescription is essentially consultations with PCPs [32].

For people with T2DM, different clinically important variables regarding diabetes were collected at the observational period end date. These variables included age, sex, body mass index (BMI), blood pressure (diastolic and systolic blood pressure), and comorbidities (history of stroke, peripheral artery disease, heart failure, ischemic heart disease, dyslipidemia, hypertension, and diabetic complications). To identify the clinically important comorbidities, we searched for documented ICD10 diagnostic codes for these conditions. Moreover, the variables related to the visits (number of visits performed by the PCP or nurse and number of visits referred to specialists) and laboratory parameters, including HbA1c, lipid profile (LDL cholesterol and triglycerides), and determination of renal variables (glomerular filtration rate, CKD-EPI equation, and albumin-to-creatinine ratio), were collected. 

### 2.4. Statistical Methods

The demographic and working profile of the included PCPs and the demographic and clinical profile of the included subjects were described using the most appropriate statistics in each case, depending on the variable type. The categorical variables were described as numbers and frequencies, while continuous variables were described as mean and standard deviation or median and quartiles (Q1; Q3). No formal sampling method was used in the study due to the cross-sectional design and nature of the dataset; we analyzed all data available that met the study criteria during the study’s observational period.

To estimate the performance of PCPs in controlling glycemia, lipids, and blood pressure, several clinical parameters were considered. For the control of glycemia, we used the glycemic assessment by HbA1c. For the control of lipids, we used the laboratory parameters for LDL cholesterol and triglycerides, while for the control of blood pressure, we used systolic and diastolic blood pressure. The rates of patients undergoing these assessments were estimated. Bivariate analysis was performed to estimate the odds ratios (OR) and 95% confidence intervals (Cis) for the control of risk factors and clinical characteristics of the subjects. Moreover, we estimated the rates of patients achieving specific goals of control defined as follows: HbA1c < 7%, LDL cholesterol < 130 mg/dL in those without cardiovascular disease (CVD), LDL cholesterol < 100 mg/dL in those with CVD, TG < 150 mg/dL, and blood pressure values < 140/90 mmHg. The cut-off points for the target of control for the different clinical parameters were applied according to the utilized local and international guidelines for diabetes during the observational period [33,34]. The specific targets are shown in Supplementary Materials Table S1.

To determine which PCPs’ characteristics (sex of the professionals, practice size (categorized as ≥400–1000; ≥1000–1500; ≥1500–2000; ≥2000 patients), proportion of people at least 65 years old in the practice, DI of the center MEDEA, and rurality) were related to the control of glycemia, lipids, and blood pressure, we estimated the mean and the standard deviation (SD) of the proportion of subjects achieving specific goals of control. Generalized linear mixed-effects (GLME) models were performed to assess the relation between different PCP characteristics (factors for variability) and specific goals of control for glycemia alone or a combination of glycemia, lipids, and blood pressure. We utilized GLME models to capture the relationship between control and independent variables, accommodating varying coefficients across grouping variables. The GLME models are ideal for nested or clustered data structures, where observations are grouped within higher-level units, such as the frequency of laboratory analyses per year with specific health metrics. They enable the modeling of correlation and heterogeneity within clusters through random effects, enhancing model precision. The models were first adjusted for variables such as professionals’ sex, practice size, the proportion of people at least 65 years old in practice, and rurality. Then, the second model deprivation index was added to the initial adjustment variables. Due to the nature and limitations of the local indicators, they were not considered covariates for the GLME models [35,36]. Rate estimates were accompanied by a 95% CI estimated following the Clopper–Pearson approximation for a binomial distribution. Data processing and statistical analyses were carried out with the R program version 6.1.0 (The R Foundation for Statistical Computing Vienna, Austria https://cran.r-project.org/ (accessed on 21 January 2024)). Mixed models were estimated using the lme4 libraries (https://cran.r-project.org/web/packages/lme4/index.html (accessed on 21 January 2024)).

The SIDIAP database undergoes regular quality checks as part of internal data quality control protocols. The study’s conduct was governed using standard operating procedures, encompassing internal quality audits, secure data storage protocols, document archiving methods, programming quality controls, analysis plan standards, and scientific review requirements. Each program drafted by a study analyst was independently reviewed by another analyst under the supervision of a senior statistician. Key study documents, including analysis plans, abstraction forms, and reports, underwent rigorous quality control and senior scientific scrutiny.

## 3. Results

At the end of the observational period, we identified 3267 PCPs and 367,132 subjects with T2DM who met the study criteria. Supplementary Materials Figures S1 and S2 show the flowchart of the study.

### 3.1. Characteristics of PCPs and Subjects with T2DM

Regarding PCPs, the majority were females (63.1%), the mean ± SD size of the assigned number of patients (practice size) was 1512 ± 313, and the mean ± SD proportion of patients aged 65 years or older across the healthcare practices was 21.7 ± 6.5%. Regarding healthcare quality standards (EQA), we found a mean value of 760 out of 1000 throughout 2017. The observed global Pharmaceutical Prescribing Quality Standard (EQPF) was 61.2 out of 130. The characteristics of primary care practices are summarized in Supplementary Materials, Table S2. 

Most subjects with T2DM were males, with a mean ± SD age of 70 ± 12.2 years, a mean ± SD BMI of 30.2 ± 5.2 kg/m^2^, and a median diabetes duration of 8.8 years. Regarding the comorbidities, hypertension was the most prevalent condition (72.1%), followed by dyslipidemia (69.3%), and cardiovascular disease (21.2%). The mean ± SD of HbA1c was 7.07% ± 1.3. Most subjects with T2DM were cared for by PCPs or nurses, and only 5% had been seen by a specialist during the study period. Annually, the average number of visits was 21 and 20 with PCPs and nurses, respectively. Table 1 summarizes the characteristics of people with T2DM and the number of PCP contacts.

### 3.2. Glycemia, Lipids, and Blood Pressure Control in Subjects with T2DM

During the 12-month observation period, 80.1% of subjects with T2DM had at least one laboratory assessment. A total of 76.1% of subjects had an HbA1c value assessment, and 78% had a lipid profile assessment. Blood pressure was measured in 83.5% of subjects. 

A total of 42.6% of subjects achieved HbA1c < 7%. The goal of control for TG (<150 mg/dL) was achieved in 63.5% of subjects. Target achievement for LDL (<130 mg/dL) for those without CVD was 55.8%, while target LDL (<100 mg/dL) in those with CVD was 34.3%. Regarding the blood pressure target (<140/90 mmHg), this was achieved by 59.2% of individuals. More detailed results are shown in Table 2. 

In the bivariate analysis, the presence of comorbidities was positively associated with a higher rate of HbA1c control, lipid profile control, or blood pressure control. On the other hand, the presence of heart failure was negatively associated with HbA1c assessment. Male sex was negatively associated with the assessment of all three risk factors. Table 3 shows the bivariate analysis of the control of different risk factors and characteristics in subjects with T2DM. 

### 3.3. Variability among PCPs in the Control of Glycemia, Lipids, and Blood Pressure

Target risk factor control for all three variables (HbA1c, lipids, and blood pressure) was stratified for different PCP’s characteristics. Female PCPs had a higher rate of target LDL achievement in subjects with CVD and target blood pressure values < 140/90 mmHg. Regarding practice size, large practices (≥2000 subjects assigned) showed lower target achievement rates for all variables except for blood pressure control, which remained at 82.5%, similar to the rest of the practice sizes. Regarding the proportion of older subjects in a given practice, those with a proportion of ≥18–25% of patients 65 years of age or older showed improved control for most of the indicators, except for target HbA1c and determination of kidney-related variables. A higher achievement rate of target HbA1c was found in practices with a proportion of ≥25% of patients aged 65 years or older. Regarding deprivation levels, PCPs from the least deprived areas showed a higher rate of target achievement for most health control parameters, except for LDL targets in subjects with CVD. When comparing urban and rural centers, we found a higher rate of target achievement for most health parameters in semi-urban centers. A more detailed overview of the PCPs’ characteristics and goals of control for different parameters can be found in Table 4. 

In the multivariable analysis for control of glycemia alone (HbA1c < 7%), a positive association was found with female PCPs, a practice size of ≥1000–1500 patients, a higher proportion of subjects with T2DM of ≥65 years, and being professionals from semi-urban or rural primary care centers. In contrast, centers with both the highest and lowest deprivation index showed a negative association with HbA1c target achievement. On the other hand, for the combined risk factor control (HbA1c < 7%, blood pressure < 140/90 mmHg, LDL < 130 mg/dL in those without CVD, LDL < 100 mg/dL in those with CVD, and TG < 150 mg/dL), a practice size of ≥1000–1500 and a proportion of people with T2DM ≥ 65 years were positively associated. Being a professional from a rural center or least-deprived area was negatively associated with the combined risk factor control. Supplementary Figure S3 and Table 5 show the multivariable models for different objectives of control.

## 4. Discussion

Our study shows a significant variability among PCPs in their performance in the management of glycemia, lipids, and blood pressure among the people with T2DM in Catalonia. Moreover, several PCP-related factors were found to be associated with performance in achieving specific target control for these risk factors. Practice size, a higher proportion of patients aged 65 years or older, and rurality were found to be common factors associated with variability in performance. 

Based on evidence reported in recent years, improving glycemic control remains an important issue for many people with T2DM and their clinicians, despite the availability of multiple therapeutic options. In our analysis, we observed that although 76.1% of the people with T2DM in our study had HbA1c assessments in the previous year, only 42.6% achieved an HbA1c value below 7% (53.0 mmol/mol). The percentage of people achieving this target HbA1c in our study is lower than reported in other international or national studies [11,37,38,39,40,41]. 

Different studies have tried to evaluate the variability in performance among PCPs regarding the recommended procedures for the care of people with T2DM. The results of these studies differ from country to country and according to the methodology used; however, they generally report that there is variation between PCPs in their performance of care processes. More specifically, a study conducted with Norwegian primary healthcare providers by Noklevy et al. [42] showed a large variability between PCPs in the performance of diabetes care processes; these variations were most strongly associated with factors reflecting structure (e.g., use of a standardized follow-up form was associated with better performance), while a heavy PCP workload and older PCP age were associated with poorer performance. In our study, we observed that female professionals, smaller practice sizes (between 1000 and 2000 patients), having a larger proportion of patients ≥ 65 years old, and being from semi-urban or rural centers were found to be factors associated with better performance for achieving HbA1c targets, while a higher deprivation index showed an association with poorer glycemic control. Similar results regarding PCP gender were observed in a Hungarian cross-sectional study [43], whereby female professionals were an independent predictor of better glycemic control. Furthermore, female general practitioners have been reported to perform better in reaching blood pressure and lipid treatment goals [44,45,46,47]. Gender differences in practice styles have been reported [48,49] or in the types of patients seen [50]. The reason why the gender factor is related to better control should be evaluated more specifically and comprehensively to identify the specific factors responsible for the difference (educational, cultural, social, or biological) [51]. Usually, these factors are hard to evaluate in a real-world analysis due to the nature of the dataset, which comes from routinely collected healthcare databases, where these variables are often not collected. Regarding practice size, a systematic review and meta-analysis by Riordan et al. published in 2020 [52] reported that the results of a relation between practice size and actual quality of care were inconsistent, although larger practices were associated with more structured care. The reason for a smaller practice size being associated with better performance in our study should be investigated more, and differences in results could be due to the specific characteristics of the primary healthcare system in each country or due to different variables within the medical consultation (e.g., spending more time with patients, continuity of care, knowing the patient better) [53]. 

Thus far, different strategies have been reported to improve diabetes care between different healthcare professionals, showing interventions targeting the chronic disease management system along with patient-mediated quality improvement strategies as important components for improving diabetes management. In contrast, interventions solely targeting healthcare professionals through quality improvement strategies might be beneficial only if baseline HbA1c control is poor [54]. The results of a comprehensive healthcare intervention centered on healthcare professionals and individuals with T2DM to reduce therapeutic inertia designed by our group in primary healthcare centers showed that maintaining good glycemic control was only achieved in the intervention group by performing periodic follow-up visits with proper control of blood metabolic parameters and treatment [55]. In addition, a meta-analysis has found that team-based care in diabetes improved blood glucose, hypertension, and lipid levels [56], which might be interesting to note for further studies when evaluating variability in different healthcare settings.

Concerning the achievements in the control of the other CVD risk factors (lipid profile and blood pressure), our results highlight that only a third of the subjects in our primary healthcare centers with a history of CVD achieved the LDL targets. Target blood pressure achievement (<140/90 mmHg) occurred in 59.2% of subjects. These data are lower compared with previously published data from our group regarding the control of blood pressure and LDL-cholesterol with CVD achieved during 2017 [11], which could be due to the different study criteria. On the other hand, compared with a previous study performed in Spain evaluating the prevalence and control of T2DM among primary care physicians in 2017 [57], the authors reported similar findings for blood pressure control (87.5%) as well as for LDL in those without CVD (88.6%). Moreover, that study reported that only 42.9% of subjects with CVD achieved good control of LDL. The average practice size in our study was smaller compared to the practice size in another national study (1512 ± 313 versus 1644.4 ± 701.4 subjects in the quota) [56]. We found a positive association between practice sizes (≥1000–1500 and ≥1500–2000 people) and good control of the combination of all risk factors. However, the size of the practice as a factor in the quality of care remains controversial. A study from the U.K. reported no differences in quality of care between practices with a larger number of patients and those with fewer patients per doctor [58]. 

Our study has some limitations. Firstly, due to the nature of the professional’s database (administrative routinely collected information on the healthcare professionals), there was insufficient information related to the PCPs (age, health problems, experience years) or variables related to the problems, barriers perceived by healthcare professionals, or other variables (educational, cultural, workload, or specific responsibilities within the centers or healthcare team) that might contribute to these results. Secondly, the data analyzed comes from real-world settings, and missing values for some variables were present. Additionally, as a limitation, we believe that a more extensive prospective follow-up study could be performed, providing health professionals with questionnaires that shed some light on the matter. Target HDL values were not included in the analysis, as this is not a modifiable variable through available drug interventions. We did not use customized target values for older adults (aged ≥ 80 years) or those with other comorbidities. The local indicators were not used in the multivariable analysis since they are highly variable and change from year to year, prioritizing the financial impact and process compliance criteria, which fail to align with the principles of universal accessibility to healthcare or the benefits of the drug in the prevention of comorbidities and mortality [36]. Moreover, inferences about causality are also limited by the study design. The strength of this study is the availability of extensive real-world data that comes from using a well-validated primary healthcare database for research on diabetes, which routinely includes data from the largest healthcare provider in Catalonia, Spain. This study was performed with data from over 250 primary healthcare providers in Catalonia, with almost forty million visits recorded annually. Over 350,000 people with T2DM and over 3000 healthcare professionals were included. This ensures a broad representation of both the subjects with T2DM and professionals in this population.

## 5. Conclusions

In our primary healthcare setting in Catalonia, we observed variability in achieving control targets for glycemia, blood pressure, and lipids among individuals with T2DM. Less than half of the subjects attained glycemic control targets, while only a third of those with a previous history of CVD reached the LDL targets. Target blood pressure was achieved in more than half of the subjects. Female PCPs, smaller practice sizes (1000–1500 people), a higher proportion of patients aged ≥ 65 years, and rural practices were positively associated with the achievement of target control for these variables. Standardizing and appropriately distributing primary care medical practices based on size and the proportion of older adults could have benefits for more effective health management of individuals with diabetes. Further research investigating how these factors contribute to variability is warranted. By ensuring proper control of these factors among healthcare professionals, we could enhance the primary care system and improve the management and control of diabetes and other chronic diseases.

## Figures and Tables

**Table 1:** Characteristics of the subjects with T2DM and the number of PCP contacts.

Variable	T2DM n = 367,132
Sociodemographic	
Sex, (female), n (%),	166,175 (45.3)
Age, (years), mean (SD)	70.0 (12.2)
Clinical variables	
BMI, (kg/m^2^), mean (SD)	30.2 (5.2)
Systolic blood pressure, (mmHg), mean (SD)	133 (13.8)
Diastolic blood pressure, (mmHg) mean (SD)	75.0 (9.8)
Comorbidities	
Diabetes duration, median (Q1; Q3)	8.81 (4.94; 13.2)
Stroke, n (%)	35,141 (9.6)
Peripheral artery disease, n (%)	22,695 (6.2)
Ischemic heart disease, n (%)	32,196 (8.8)
Hearth failure, n (%)	27,281 (7.4)
Cardiovascular disease, n (%)	77,731 (21.2)
Diabetic retinopathy, n (%)	37,424 (10.2)
Dyslipidemia, n (%)	254,422 (69.3)
Hypertension, n (%)	264,865 (72.1)
Laboratory parameters, mean, SD	
HbA1c, %	7.07 (1.3)
LDL, mg/dL	103 (32.9)
Total cholesterol, mg/dL	183 (40.2)
TG, mg/dL	160 (106)
Estimated glomerular filtration rate (ml/min/1.73 m^2^)	69.5 (18.0)
Urine albumin to creatinine ratio (mg/L)	60.6 (222)
Annual medical visits	
PCP, n (%)	362,370 (99.7)
Number of PCP visits, mean (SD)	21.4 (14.6)
Nurse, n (%)	357,993 (98.5)
Number of nurse visits, mean (SD)	20.0 (22.2)
Specialist, n (%)	18,553 (5.05)
Number of specialists visits, mean (SD)	1.16 (0.5)

T2DM: type 2 diabetes mellitus; BMI: body mass index; SD: standard deviation; HbA1c: glycated hemoglobin; LDL: low-density lipoprotein; TG: triglycerides; N: number; PCP: primary care physician.

**Table 2:** Control of glycemia, lipids, and blood pressure during the study period.

Risk Factor Control	Number of Events	Rates (%)	Lower 95% CI	Upper 95% CI
Any laboratory assessment	294,046	80.1	79.9	80.2
**Glycemic control**				
HbA1c determinations	279,523	76.1	76.0	76.3
HbA1c < 7%	156,501	42.6	42.5	42.8
**Lipid profile control**				
Lipid profile determination	286,481	78.0	77.9	78.2
LDL determination	254,303	69.3	69.1	69.4
LDL < 130 mg/dL without CVD	204,839	55.8	55.6	55.9
LDL < 100 mg/dL with CVD	125,997	34.3	34.2	34.4
TG determination	272,165	74.1	73.9	74.3
TG < 150 mg/dL	233,119	63.5	63.3	63.7
**Blood pressure control**				
Blood pressure assessment	306,560	83.5	83.4	83.6
Blood pressure < 140/90 mmHg	217,636	59.2	59.1	59.4

CI: confidence interval; HBA1c: glycated hemoglobin; LDL: low-density lipoprotein; TG: triglycerides.

**Table 3:** Bivariate analysis of glycemic, lipid, and blood pressure control with different characteristics associated with diabetes.

Variables	HbA1c Control ^1^	Lipid Control ^2^	Blood Pressure Control ^3^
	OR (95% CI)	OR (95% CI)	OR (95% CI)
Male, ref: Female	0.86 (0.85, 0.88)	0.83 (0.81, 0.84)	0.87 (0.86, 0.89)
Stroke	0.99 (0.97, 1.01)	1.09 (1.06, 1.12)	1.32 (1.28, 1.37)
Peripheral artery disease	1.11 (1.08, 1.15)	1.15 (1.11, 1.19)	1.67 (1.60, 1.74)
Ischemic heart disease	1.05 (1.02, 1.08)	1.15 (1.12, 1.18)	1.68 (1.62, 1.74)
Heart failure	0.94 (0.91, 0.96)	1.09 (1.06, 1.13)	2.11 (2.02, 2.20)
Cardiovascular disease	1.05 (1.03, 1.07)	1.14 (1.12, 1.17)	1.55 (1.52, 1.59)
Diabetic retinopathy	1.33 (1.29, 1.36)	1.21 (1.18, 1.25)	1.47 (1.43, 1.52)
Dyslipidemia	1.19 (1.17, 1.21)	1.22 (1.20, 1.24)	1.26 (1.23, 1.28)
Hypertension	1.33 (1.31, 1.35)	1.40 (1.38, 1.43)	1.90 (1.86, 1.93)

CI: confidence interval; HbA1c: glycated hemoglobin; OR: odds ratio; T2DM: type 2 diabetes mellitus; ref: in reference to. ^1^ HbA1c control: HbA1c < 7%. ^2^ Lipid control: LDL < 130 mg/dL without CVD, LDL < 100 mg/dL with CVD, TG < 150 mg/dL. ^3^ Blood pressure control: blood pressure < 140/90 mmHg.

**Table 4:** PCP-related characteristics and control goals for glycemia, lipids, and blood pressure.

PCP-Related Variables	HbA1c < 7%	LDL < 130 mg/dL in Those with CVD	LDL < 100 mg/dL in Those with CVD	TG < 150 mg/dL	Blood Pressure < 140/90 mmHg
**Sex, mean, (SD)**					
Female	42.5 (7.8)	42.2 (8.5)	10.4 (3.9)	63.4 (9.5)	59.5 (10.1)
Male	41.9 (8.3)	42.3 (8.7)	10.1 (3.6)	62.9 (9.5)	59.0 (11.1)
**Practice size, (Number of patients), mean, (SD)**					
≥400–1000	43.4 (9.6)	44.0 (11.3)	10.1 (4.3)	64.8 (10.7)	58.2 (12.4)
≥1000–1500	42.8 (7.9)	42.7 (8.4)	10.4 (3.9)	64.2 (9.1)	59.0 (10.7)
≥1500–2000	42.3 (7.5)	42.2 (7.9)	10.3 (3.6)	63.2 (8.9)	59.6 (10.1)
≥2000	36.9 (8.9)	35.1 (10.6)	8.8 (3.8)	53.8 (13.3)	59.7 (9.6)
**Proportion of patients ≥ 65 years old, mean, (SD)**					
≥2–18	41.4 (7.9)	42.3 (8.2)	9.8 (3.8)	63.1 (9.1)	59.6 (10.3)
≥18–25	42.5 (7.7)	42.5 (8.1)	10.6 (3.7)	63.6 (9.1)	59.6 (10.4)
≥25	43.1 (8.4)	41.9 (9.8)	10.2 (3.8)	63.0 (10.4)	58.5 (10.8)
**MEDEA, mean, (SD)**					
Least deprived	43.5 (9.4)	44.1 (9.8)	9.6 (4.2)	64.9 (9.6)	57.5 (12.3)
Most deprived	42.7 (7.1)	42.7 (8.2)	10.3 (3.6)	63.6 (9.1)	59.7 (10.4)
Missing	44.3 (4.9)	35.2 (7.2)	7.5 (2.7)	54.0 (8.6)	63.5 (8.2)
**Rurality, mean, (SD)**					
Urban center	41.7 (7.8)	41.3 (8.4)	10.5 (3.8)	62.3 (9.5)	59.7 (10.1)
Semi-urban center	43.4 (8.2)	45.6 (7.8)	11.0 (3.9)	67.4 (8.7)	59.7 (10.8)
Rural center	43.0 (7.9)	42.4 (8.9)	9.4 (3.6)	63.0 (9.2)	58.3 (10.8)

CVD: cardiovascular disease; HbA1c: glycated hemoglobin; LDL: low-density lipoprotein; MEDEA: deprivation index; TG: triglycerides.

**Table 5:** Multivariable models of PCP-related variables and control of glycemia (HbA1c <7%) and control for the combination of risk factors.

	HbA1c < 7%		^1^ Control for Combined Risk Factors	
Factors for Variability	Estimates (CI)	Estimates (CI)	Estimates (CI)	Estimates (CI)
(Intercept)	0.78 (0.69; 0.89)	0.90 (0.73; 1.10)	0.01 (0.00; 0.01)	0.01 (0.00; 0.01)
Female professional	1.03 (1.01; 1.06)	1.03 (1.01; 1.06)	1.00 (0.94; 1.07)	1.02 (0.96; 1.08)
Practice size, (patients), ≥400–1000 *	0.43 (0.32; 0.58)	0.44 (0.33; 0.59)	1.19 (0.50; 2.81)	1.57 (0.66; 3.72)
Practice size, (patients), ≥1000–1500 *	1.73 (1.48; 2.03)	1.72 (1.46; 2.03)	16.90 (10.82; 26.40)	12.91 (8.14; 20.45)
Practice size, (patients), ≥1500–2000 *	0.43 (0.35; 0.54)	0.46 (0.37; 0.58)	1.43 (0.78; 2.62)	2.22 (1.20; 4.10)
Proportion of subjects over 65 years	1.00 (1.00; 1.01)	1.01 (1.00; 1.01)	1.03 (1.02; 1.03)	1.03 (1.03; 1.04)
Semi-urban centre **	1.07 (1.04; 1.10)	1.05 (1.02; 1.08)	0.99 (0.91; 1.08)	0.94 (0.86; 1.02)
Rural centre **	1.05 (1.02; 1.08)	1.01 (0.97; 1.04)	0.81 (0.75; 0.87)	0.80 (0.73; 0.87)
Least deprived area ***		0.80 (0.68; 0.94)		0.50 (0.34; 0.76)
Most deprived area ***		0.83 (0.71; 0.97)		0.69 (0.46; 1.04)
Observations	3200	3200	3200	3200
R^2^	0.044	0.059	0.035	0.039
AIC	−7228.026	−7267.598	−15,390.124	−15,433.925

AIC: Akaike information criterion (negative AIC indicates less information loss than a positive AIC and therefore a better model); CI: confidence interval; HbA1c: glycated hemoglobin; R^2^: coefficient of determination; Ref: in reference to; ^1^ Control for the combination of risk factors (HbA1c < 7%, blood pressure < 140/90 mmHg, LDL < 130 mg/dL without CVD, LDL < 100 mg/dL with CVD, TG < 150 mg/dL); * Ref: ≥2000; ** Ref: urban center; *** Ref: missing.

## Data Availability

Due to the legal limitations, the dataset of this study is a property of the DAP_Cat group. It could be requested from the corresponding author, Josep Franch-Nadal, via email at josep.franch@gmail.com.

## Supplementary Materials

The following supporting information can be downloaded at: https://www.mdpi.com/article/10.3390/jcm13061544/s1. Figure S1. Flowchart selection criteria for subjects; Figure S2. Flowchart selection criteria for primary care physicians; Supplementary Table S1. Specific goals of control for glycemia, lipids, and blood pressure; Supplementary Table S2. Characteristics of the primary healthcare practices; Figure S3. Multivariable models of PCP-related variables and control of glycemia, lipids, and blood pressure [59,60].
