# Supplementary material for: Performance of Primary Care Physicians in the Management of Glycemia, Lipids, and Blood Pressure among People with Type 2 Diabetes: A Cross-Sectional Study
Source: J Clin Med. 2024 Mar 7;13(6):1544. doi: 10.3390/jcm13061544 (PMC10970782; doi:10.3390/jcm13061544)
Supplement: Supplementary file 1 [file jcm-13-01544-s001.zip › jcm-2861079-supplementary.pdf]

Supplementary material

Variability among primary care physicians in the management of people with type 2 diabetes: a cross-sectional study

Bogdan Vlacho[1,2,3+], Berta Fernandez-Camins[1,2+], Albert Canudas-Ventura[4,5], Andrés Rodríguez[6], Àngels Mollo[1,6], Francesc Xavier Cos[1,7], Maria Antentas[2], Dídac Mauricio[1,2,3,8], Josep Franch-Nadal[*1, 3, 9]

Title	page
Figure S1. Flowchart selection criteria subjects	1
Figure S2. Flowchart selection criteria for primary care physicians	1
Table S1. Specific goals of control for glycemia, lipids and blood pressure	3
Table S2. Characteristics of the primary healthcare practices	4
Figure S3. Multivariable models of PCP-related variables and control of glycemia, lipids and blood pressure	5

Figure S1. Flowchart selection criteria subjects

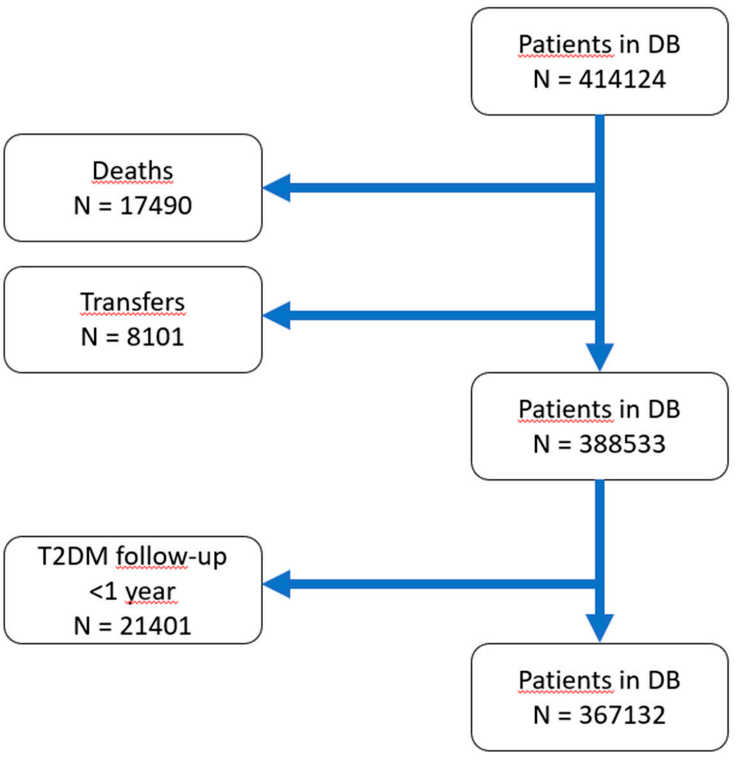

Figure S2. Flowchart selection criteria for primary care physicians

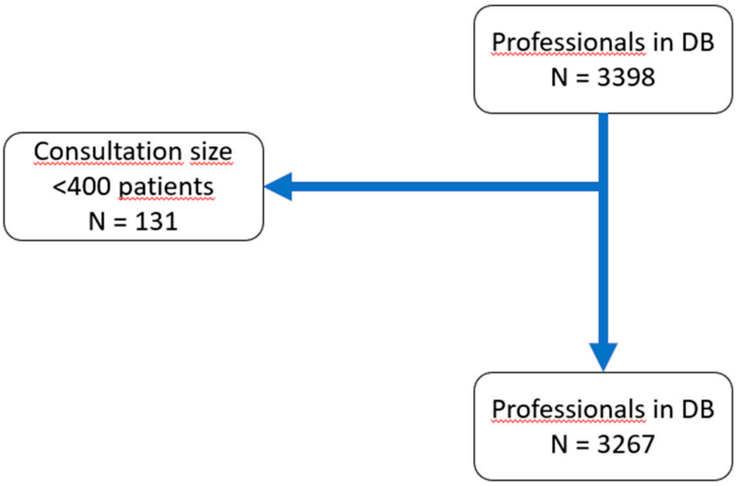

DB: data base.

Table S1. Specific targets of control for glycemia, lipids and blood pressure

Clinical variable	Objective of control
HbA1c	< 7 %.
LDL cholesterol	< 130 mg/dL without CVD.<br>< 100 with CVD.
Triglycerides	< 150 mg/dL.
sBP	< 140 mmHg.
dBP	< 90 mmHg.

HbA1c: glycated haemoglobin. LDL: low density lipoprotein. CVD: cardiovascular disease. CKD-EPI: Rate estimated through the Chronic Kidney Disease Epidemiology Collaboration equation. sBP: systolic blood pressure. dBP: diastolic blood pressure.

Table S2. Characteristics of the primary care practices

Variable	2017	Number
**Sex of the PCP (female), n (%)**	2063 (63.1)	3267
**Practice size,** mean (SD)	1512 (313)	3267
**Patients aged ≥65 years,** percentage mean (SD)	21.7 (6.51)	3267
**Male patients in the practice,** mean (SD)	49.0 (3.41)	3267
**EQA***, Mean (SD)	760 (100)	3266
Median [Q1;Q3]	772 [716;824]	3266
**EQPF****, Mean (SD)	61.2 (18.8)	3046
Median [Q1;Q3]	63.0 [49.0;75.0]	3046

PCP: primary healthcare physician; * EQA interpretation aid; ** EQPF interpretation aid

EQA interpretation aid

The healthcare quality standard was constructed to serve as a synthetic indicator to measure the quality of care provided by primary care professionals in Catalonia (Spain). It involves over 60 indicators and is calculated on a monthly basis using an automated process that extracts data from electronic medical records. It assigns a global score on a scale of 0 to 1000 points. The results are compared with minimum and maximum targets established in January of each year, based on the 20th and 80th percentiles of the results over the previous 12 months. In addition, each primary care professional or team has an individual target on the synthetic indicator that is drawn from the baseline score and rewards efforts to improve. [1]

A detailed description of each indicator included in EQA evaluation can be found at: http://www.camfic.formulari.cat/DemoECAP/umi_1/sisap-umi.eines.portalics/indicador/indicador/1303/ver/index.html

** EQPF interpretation aid

The EQPF was designed to incentivize better prescription of drugs in primary care practice. The quality criteria and indicators are supported by scientific evidence [2] and are designed for multidisciplinary teams with the aim of increasing the use of the most cost-effective drugs and ultimately reducing the enormous variability associated with the treatment of the most common diseases dealt with in primary care. [3]

For each indicator, a numerical target is scored based on the values of the best positioned GPs in all Catalonia. That is, based on the score obtained by professionals who prescribe more appropriately, the scores for evaluating all professionals are established. The achievement of each target is scored and weighted according to its relevance and difficulty. The scores for each section are added up and the total possible score for the area of family and community medicine ranges from 0 to 100 [3].

A detailed description of each indicator included in EQPF evaluation can be found at: https://ics.gencat.cat/web/.content/Documents/assistencia/EQPF-2022-GLOBAL-i-MFiC-V.2.pdf

References

1. Coma E, Ferran M, Méndez L, Iglesias B, Fina F, Medina M. Creation of a synthetic indicator of quality of care as a clinical management standard in primary care. Springerplus. 2013;2(1):1–11.

2. Gilabert-Perramon A, López-Calahorra P, Escoda-Geli N, Salvadó-Trias C. Receta electrónica en Cataluña (Rec@t): una herramienta de salud. Med Clin (Barc). 2010 Jan;134(SUPPL. 1):49–55.

3. Yuguero O, Marsal JR, Esquerda M, Galvan L, Soler-González J. Cross-sectional study of the association between empathy and burnout and drug prescribing quality in primary care. Prim Health Care Res Dev. 2019 Oct 30;20:e145.

Figure S3. Multivariable models of PCP-related characteristics and achievement of different target. A) HbA1c target (<7%) achievement and PCP´s characteristics: sex, practice size, proportion of patients ≥65 years old and rurality. B) HbA1c target (<7%) achievement and PCP´s characteristics: sex, practice size, proportion of patients ≥65 years old, rurality and deprivation index. C) Combined risk factor target achievement* and PCP´s characteristics: sex, practice size, proportion of patients ⩾65 years old and rurality.D) Combined risk factor target achievement* and PCP´s characteristics: sex, practice size, proportion of patients ⩾65 years old, rurality and deprivation index. Combined risk factor target achievement (HbA1c <7%, blood pressure<140/90 mmHg; LDL <130 mg/dL for subjects without CVD or LDL<100 mg/dL for subjects with CVD; and TG <150 mg/dL,)

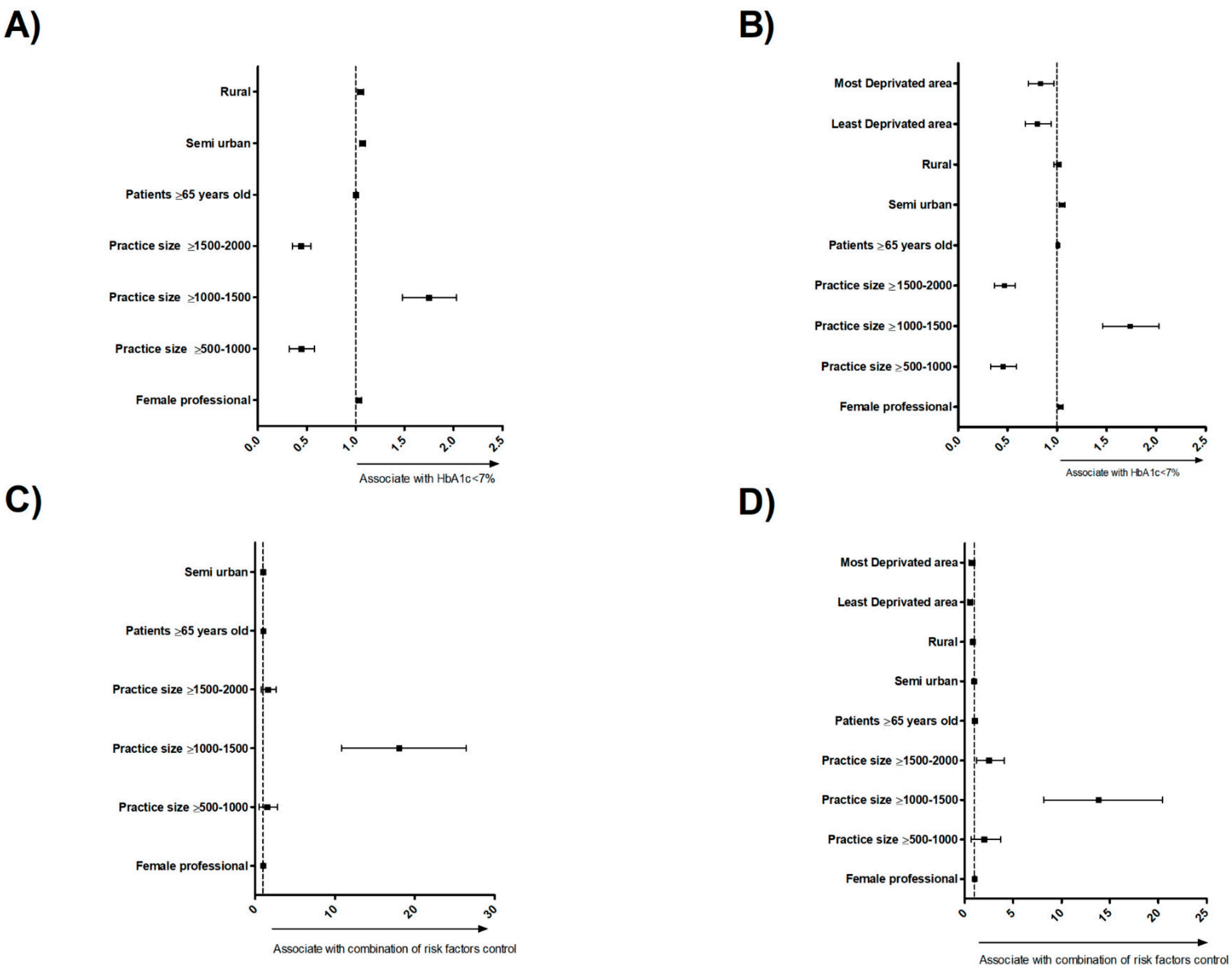
A)
Rural
Semi urban
Patients ≥65 years old
Practice size ≥1500-2000
Practice size ≥1000-1500
Practice size ≥500-1000
Female professional
0.0
0.5
1.0
1.5
2.0
2.5
Associate with HbA1c<7%
B)
Most Deprivated area
Least Deprivated area
Rural
Semi urban
Patients ≥65 years old
Practice size ≥1500-2000
Practice size ≥1000-1500
Practice size ≥500-1000
Female professional
0.0
0.5
1.0
1.5
2.0
2.5
Associate with HbA1c<7%
C)
Semi urban
Patients ≥65 years old
Practice size ≥1500-2000
Practice size ≥1000-1500
Practice size ≥500-1000
Female professional
0
10
20
30
Associate with combination of risk factors control
D)
Most Deprivated area
Least Deprivated area
Rural
Semi urban
Patients ≥65 years old
Practice size ≥1500-2000
Practice size ≥1000-1500
Practice size ≥500-1000
Female professional
0
5
10
15
20
25
Associate with combination of risk factors control
